# Impact of Point-of-Care Ultrasound on Prehospital Decision Making by HEMS Physicians in Critically Ill and Injured Patients: A Prospective Cohort Study

**DOI:** 10.1017/S1049023X23006003

**Published:** 2023-08

**Authors:** Niek J. Vianen, Esther M.M. Van Lieshout, Koen H.A. Vlasveld, Iscander M. Maissan, Patricia C. Gerritsen, Dennis Den Hartog, Michael H.J. Verhofstad, Mark G. Van Vledder

**Affiliations:** 1. Trauma Research Unit Department of Surgery, Erasmus MC, University Medical Center Rotterdam, Rotterdam, The Netherlands; 2. Erasmus University Medical Center Rotterdam, Department of Anesthesiology, Rotterdam, The Netherlands; 3. Department of Intensive Care, Erasmus University Medical Center, Rotterdam, The Netherlands

**Keywords:** decision making, Emergency Medical Services, emergency medicine, prehospital, ultrasound

## Abstract

**Introduction:**

Several studies have shown the additional benefit of point-of-care ultrasound (POCUS) by prehospital Emergency Medical Services (EMS). Since organization of EMS may vary significantly across countries, the value of POCUS likely depends on the prehospital system in which it is used. In order to be able to optimally implement POCUS and develop a tailored training curriculum, it is important to know how often POCUS is currently used, for which indications it is used, and how it affects decision making. The aims of this study were: (1) to determine the percentage of patients in whom POCUS was used by Dutch Helicopter Emergency Medical Services (HEMS) crews; (2) to determine how often POCUS findings led to changes in on-scene management; and (3) what these changes were.

**Methods:**

Patients who received prehospital care from December 1, 2020 through March 31, 2021 by a single HEMS crew were included in this prospective cohort study. Clinical data and specific data on POCUS examination, findings, and therapeutic consequences were collected and analyzed.

**Results:**

During the study period, on-scene HEMS care was provided to 612 patients, of which 211 (34.5%) patients underwent POCUS. Of these, 209 (34.2%) patients with a median age of 45 years were included. There were 131 (62.7%) trauma patients, and 70 (33.7%) of the included patients underwent cardiopulmonary resuscitation (CPR). The median reported time of POCUS examination was three (P_25_-P_75_ 2-5) minutes. Median prolongation of on-scene time was zero (P_25_-P_75_ 0-1) minutes. In 85 (40.7%) patients, POCUS examination had therapeutic consequence: POCUS was found to impact treatment decisions in 34 (26.0%) trauma patients and 51 (65.4%) non-trauma patients. In patients with cardiac arrest, POCUS was most often used to aid decision making with regard to terminating or continuing resuscitation (28 patients; 13.4%).

**Conclusion:**

During the study period, POCUS examination was used in 34.5% of all prehospital HEMS patients and had a therapeutic consequence in 40.7% of patients. In trauma patients, POCUS seems to be most effective for patient triage and evaluation of treatment effectiveness. Moreover, POCUS can be of significant value in patients undergoing CPR. A tailored HEMS POCUS training curriculum should include ultrasound techniques for trauma and cardiac arrest.

## Introduction

Point-of-care ultrasound (POCUS) has been widely used in prehospital management of severely ill and injured patients in many prehospital systems across the globe.^1^ As prehospital POCUS can provide the health care provider with important additional information, it is thought to aid decision making and improve quality of care and outcomes.

In a 2011 consensus paper, the use of prehospital ultrasound was ranked among the top five research priorities in physician-based prehospital critical care, and three key research questions were formulated: (1) Which ultrasound examinations can be safely transferred to the prehospital setting? (2) How does prehospital ultrasound affect patient management and patient pathways? and (3) How should providers achieve and maintain specific ultrasound skills?^2^ In an attempt to answer these questions, Botker, et al performed a systematic review of 27 articles pertaining to prehospital POCUS, concluding that POCUS is feasible in patients with trauma and breathing difficulties, and that its use in cardiac arrest may be feasible if it does not prolong pauses in compressions.^1^ Likewise, another systematic review showed that POCUS may change patient management in up to 48.9% of trauma patients, resulting in better prehospital triage and preventing unnecessary prehospital procedures.^3^ Other studies have shown the added value of POCUS in patients with a variety of medical issues and patients with out-of-hospital cardiac arrest.^4,5^

However, the way Emergency Medical Services (EMS) are organized may vary significantly between countries, and the value of POCUS likely depends on the prehospital system in which it is used. For instance, the presence of a physician on-scene, distance to the nearest hospital, and level of training of EMS providers may all impact the way POCUS is employed and its efficacy. To be able to implement POCUS in any specific prehospital system, it is important to know how often POCUS is currently used, for which indications it is used, and how it affects decision making. Two studies have examined the role of prehospital ultrasound by Helicopter EMS (HEMS); however, some reporting bias may have occurred due to the retrospective nature of these studies.^6,7^

In the Netherlands, POCUS is mainly used by HEMS crews. An HEMS crew consists of an HEMS physician (anesthesiologist or trauma surgeon), a specialized nurse, and a pilot. All Dutch HEMS physicians are trained in chest and abdominal ultrasound according to the extended Focused Assessment with Sonography in Trauma (eFAST). In addition, most HEMS physicians have had some form of cardiac ultrasonography training. Most often, these courses have been developed for ultrasound examination in the emergency department or intensive care unit. The question is, therefore, whether the curriculum that is taught in these courses fits the prehospital needs for HEMS. For this study, it was hypothesized that a POCUS course tailored to the needs of Dutch HEMS physicians, combining eFAST with cardiac ultrasound, using portable devices, may improve ultrasound skills specifically needed in the prehospital environment. To develop such a curriculum, detailed information about current POCUS use is needed. Therefore, the aims of this study were to determine the percentage of patients in whom POCUS is used by HEMS crews, what the findings were, and how POCUS findings changed the on-scene decision making.

## Methods

### Patient Selection

This study was a prospective cohort study which included all patients from a single Dutch HEMS station serving approximately one-third of the national population, from December 1, 2020 through March 31, 2021. This included both adult and pediatric patients, and trauma and non-trauma cases. The HEMS crew was dispatched according to national guidelines and all patients that received prehospital care by the HEMS crew were included in the study. There were no exclusion criteria for participation, however, patients with incomplete case report data were excluded from the analysis.

### Data Collection and Registration

On-scene care was carried out as usual with or without POCUS at the discretion of the attending HEMS physician. Several portable ultrasound devices were used during the study period: the SonoSite M-turbo portable ultrasound machine (FIJIFILM; Bothell, Washington USA), the SonoSite Edge II portable ultrasound machine, and the Butterfly handheld ultrasound probe (Butterfly Network; Burlington, Massachusetts USA). Which portable ultrasound device was used depended on the availability and the discretion of the attending HEMS physician. During the study period, a custom case report form (CRF) was completed by the attending HEMS physician after every dispatch. The CRF contained general questions regarding patient characteristics, and more specific questions regarding use of POCUS such as the duration of the ultrasound examination, prolongation of the on-scene time, the location of the POCUS examination, the ultrasound device used to perform the examination, the anatomical area(s) of the patient that was or were examined using ultrasound, if a specific diagnosis or finding was found, and if this was the case, if there was any therapeutic consequence of this diagnosis or finding. Therapeutic consequences were defined in the following manner: (1) destination change; (2) drug therapy change; (3) fluid therapy change; (4) invasive procedure change; (5) cardiopulmonary resuscitation (CPR) initiation/termination; (6) evaluation of treatment effect; or (7) other.

### Data Analysis

Data analysis was performed using IBM’s Statistical Package for the Social Sciences (SPSS), Version 26 (IBM Corp.; Armonk, New York USA). Normality of continuous data was tested using the Shapiro-Wilk test. Only descriptive analysis was done. All continuous data were non-normal and are therefore shown as median with quartiles. Discrete data are shown as number and percentages. Percentages were corrected for missing data.

### Medical Ethics Review

The study protocol was exempted by the local Medical Research Ethics Committee (reference nr. MEC-2020-0881) and consent was waived.

## Results

During the study period, there were 1,227 HEMS dispatches, of which 615 (50.1%) were cancelled before the HEMS crew arrived on scene. The other 612 (49.9%) patients received on-scene treatment by the HEMS crew. The reason for dispatch was trauma in 326 (53.3%) patients and a non-traumatic medical emergency in 286 (46.7%) patients. Point-of-care ultrasound was performed in 211 (34.5%) patients. Two patients were excluded from the study due to incomplete CRFs, leaving 209 patients for the analysis.

### Patient and Ultrasound Characteristics

Of the 209 included patients assessed with ultrasound, 150 (72.1%) patients were male (Table 1). Trauma was the reason for dispatch in 131 (62.7%) patients, of whom 16 (12.2%) patients underwent CPR. Of the 78 patients with a non-traumatic reason for HEMS dispatch, CPR was performed in 54 (69.2%) patients. Median reported time of POCUS examination was three (P_25_-P_75_ 2-5) minutes. Median reported prolongation of on-scene time was zero (P_25_-P_75_ 0-1) minutes. In 209 patients, a total of 437 ultrasound examinations were performed (lungs n = 155, 35.5%; abdomen n = 124, 28.4%; and heart n = 158, 36.2%). In 69 (34.0%) patients, a SonoSite M-turbo portable ultrasound machine was used; in 60 (29.6%) patients, a SonoSite Edge II portable ultrasound machine was used; and in 74 (36.5%) patients, a Butterfly handheld ultrasound probe was used. For six patients, no device data were recorded. All ultrasound examinations were performed by 12 HEMS physicians, with the median number of examinations performed per physician being 15 (P_25_-P_75_ 10.5-20).

**Table 1.** Descriptive Statistics Ultrasounds

		All (n = 209)		
		n*		
**Age (years)**		209	45	23-64
**Sex** ^**a**^	Male	208	150	72.1%
**Reason for Dispatch**	Trauma	209	131	62.7%
	Non-Trauma		78	37.3%
**CPR** ^**a**^	Yes	208	70	33.7%
**Location Where POCUS was Performed** ^**b**^	On-Scene	209	86	41.1%
	During Patient Transport		138	66.0%
	In Emergency Department		2	1.0%
**Ultrasound Device** ^**a**^	Edge	203	60	29.6%
	M-Turbo		69	34.0%
	Butterfly		74	36.5%
**Estimated POCUS Time (minutes)**		209	3	2-5
**Estimated Prolonged On-Scene Time (minutes)**		209	0	0-1

Note: Data are shown as median (P25-P75) or as n (%). n* shows the number of patients/participants for whom data were available.

Abbreviations: CPR, cardiopulmonary resuscitation; POCUS, point-of-care ultrasound.

a Percentages are corrected for missing data

b Some patients underwent POCUS in more than one location.

### Change in Patient Management per Patient Category

Table 2 shows the therapeutic consequences of POCUS by and per patient category. In 85 (40.7%) patients, POCUS led to a change in patient management. In trauma patients, POCUS led to a change in management in 34 (26.0%) patients. Evaluation of treatment effect was the most common use of POCUS in trauma patients (n = 11; 8.4%), followed by a change in patient destination (n = 9; 6.9%) and a change in drug or fluid therapy (both n = 8; 6.1%). The decision to perform an invasive procedure was impacted by POCUS in only five patients (3.8%). In non-trauma patients, POCUS led to a change in management in 51 of 78 patients (65.4%). Initiation or termination of CPR was the most frequently occurring POCUS induced change in management in 22 (28.2%) patients, followed by changes in drug therapy (n = 19; 24.4%) and fluid therapy (n = 11; 14.1%). In patients undergoing CPR, POCUS findings resulted in a change in management in 53 of 70 patients (75.7%), most frequently being the initiation or termination of CPR (n = 28; 40.0%).

**Table 2.** Therapeutic Consequences of POCUS for Different Patient Categories

	All Patients (n = 209)	Trauma (n = 131)	Non-Trauma (n = 78)	CPR (n = 70)
**Any Therapeutic Consequence**	85 (40.7%)	34 (26.0%)	51 (65.4%)	53 (75.7%)
Patient Destination Change	16 (7.7%)	9 (6.9%)	7 (9.0%)	4 (5.7%)
Drug Therapy Change	27 (12.9%)	8 (6.1%)	19 (24.4%)	20 (28.6%)
Fluid Therapy Change	19 (9.1%)	8 (6.1%)	11 (14.1%)	11 (15.7%)
Invasive Procedure Performed/Called Off	6 (2.9%)	5 (3.8%)	1 (1.3%)	3 (4.3%)
CPR Initiated/Terminated	28 (13.4%)	6 (4.6%)	22 (28.2%)	28 (40.0%)
Evaluation of Treatment Effect	22 (10.5%)	11 (8.4%)	11 (14.1%)	13 (18.6%)
Other	16 (7.7%)	4 (3.1%)	12 (15.4%)	14 (20.0%)

Note: Data are shown as n (%).

Abbreviations: CPR, cardiopulmonary resuscitation; POCUS, point-of-care ultrasound.

### Change in Patient Management per Anatomical Area

Prehospital cardiac ultrasound resulted in a change in management in 57 (36.1%) patients (Table 3). Initiation or termination of CPR (n = 26; 16.5%), drug therapy change (n = 22; 13.9%), and fluid therapy change (n = 16; 10.1%) were the most frequently occurring changes in management after cardiac ultrasound. Pulmonary ultrasound led to a change in management in 27 (17.4%) patients. Evaluation of treatment effect (n = 10; 6.5%) and patient destination change (n = 6; 3.9%) were the most frequent changes in management after pulmonary ultrasound. Ultrasound of the abdomen led to a change in management in 20 (16.1%) patients, with patient destination change (n = 9; 7.3%) and evaluation of treatment effect (n = 6; 4.8%) being the most frequently occurring patient management changes after POCUS.

**Table 3.** Therapeutic Consequences of POCUS by Anatomical Area

	**All** **(n = 209)**	**Lungs** **(n = 155)**	**Heart** **(n = 158)**	**Abdomen** **(n = 124)**
**Any Therapeutic Consequence**	85 (40.7%)	27 (17.4%)	57 (36.1%)	20 (16.1%)
Patient Destination Change	16 (7.7%)	6 (3.9%)	5 (3.2%)	9 (7.3%)
Drug Therapy Change	27 (12.9%)	2 (1.3%)	22 (13.9%)	3 (2.4%)
Fluid Therapy Change	19 (9.1%)	1 (0.6%)	16 (10.1%)	2 (1.6%)
Invasive Procedure Performed/Called Off	6 (2.9%)	4 (2.6%)	2 (1.3%)	0 (0.0%)
CPR Initiated/Terminated	28 (13.4%)	1 (0.6%)	26 (16.5%)	1 (0.8%)
Evaluation of Treatment Effect	22 (10.5%)	10 (6.5%)	13 (8.2%)	6 (4.8%)
Other	16 (7.7%)	7 (4.5%)	6 (3.8%)	2 (1.6%)

Note: Data are shown as n (%).

Abbreviations: CPR, cardiopulmonary resuscitation; POCUS, point-of-care ultrasound.

### Focus of Ultrasound per Patient Category

In trauma patients, POCUS examination was mostly used to look for pneumothorax (n = 114; 87.0%), hemothorax (n = 95; 72.5%), intra-abdominal free fluid (n = 96; 73.3%), cardiac tamponade (n = 80; 61.1%), and left/right ventricular function and size (n = 84; 64.1%; Table 4). In non-trauma patients, the interest of the POCUS examination was more cardiac oriented, with specific interest in ventricular function and size (n = 69; 88.5%), presence of cardiac tamponade (n = 64; 82.1%), and atrial filling (n = 57; 73.1%) being the most frequently investigated topics. In patients undergoing CPR, ventricular function and size (n = 64; 91.4%), presence of cardiac tamponade (n = 56; 80.0%), and atrial filling (n = 48; 68.6%) were the most commonly investigated topics.

**Table 4.** Seven Most Common Findings, Number of Times Looked for Specific Diagnosis, or Finding per Patient Category

	**All** **(n = 209)**	**Trauma** **(n = 131)**	**Non-Trauma** **(n = 78)**	**CPR** **(n = 70)**
Pneumothorax	152 (72.7%)	114 (87.0%)	38 (48.7%)	31 (44.3%)
Hemothorax	128 (61.2%)	95 (72.5%)	33 (42.3%)	25 (35.7%)
Cardiac Tamponade	144 (68.9%)	80 (61.1%)	64 (82.1%)	56 (80.0%)
Effectivity of Thorax Compression	25 (12.0%)	7 (5.3%)	18 (23.1%)	25 (35.7%)
Atrial/Caval Filling	118 (56.5%)	61 (46.6%)	57 (73.1%)	48 (68.6%)
Left/Right Ventricular Function and Size	153 (73.2%)	84 (64.1%)	69 (88.5%)	64 (91.4%)
Intra-Abdominal Free Fluid	120 (57.4%)	96 (73.3%)	24 (30.8%)	47 (67.1%)

Note: Data are shown as n (%).

## Discussion

In this study, prehospital POCUS was used in approximately one-third of HEMS dispatches (n = 209) and led to a change in patient management in 40.7% of these patients. Trauma patients were most frequently encountered during the study period (62.7% of all patients), and POCUS led to a change in patient management in 26.0% of these patients. In non-trauma patients, POCUS led to a change in patient management in 65.4% of patients, most likely because over two-thirds of non-trauma patients were in cardiac arrest. In CPR patients, POCUS led to a change in management in up to 75.7%, most often being the decision to initiate or stop further resuscitation (40.0%).

Experiences with POCUS in patients with cardiac arrest in the current study are in line with the findings in the review by Botker, et al.^1^ For instance, in cardiac arrest patients, POCUS led to a change in management in 75.7% of patients in the current study. In more than one-half of these patients, this management change included a decision to cease resuscitation. In a 2013 retrospective cohort study from the Netherlands, POCUS frequently led to a change in management in patients with cardiac arrest, such as a decision to cease resuscitation (29% of cardiac arrest patients).^7^ Indeed, absence of mechanical cardiac activity on POCUS, combined with the absence of electrical cardiac activity, seems to have such a high positive predictive value for death during CPR, it is now frequently used to guide the decision to terminate (or continue) prehospital resuscitation in cardiac arrest patients.^8^ In addition to the diagnosis of absence of mechanical cardiac activity, this study found that POCUS frequently leads to changes in drug and fluid therapy and is used to evaluate treatment effect and chest quality of compressions during resuscitation. While this study did not incorporate outcome data, POCUS has the potential to improve the quality of resuscitation and thus outcomes in out-of-hospital cardiac arrest in experienced hands, as reported in recent studies by Zanatta, et al and Liu, et al.^9,10^

In the current cohort, POCUS led to a change in management in 26.0% of trauma patients. While POCUS in trauma patients was used to guide treatment decisions (either drug, fluid, or invasive procedure related) less frequently than in cardiac arrest patients, final disposition of the patient was changed in 6.9% of trauma patients after POCUS. As POCUS was found not to prolong on-scene time (POCUS can often be performed simultaneously with other prehospital procedures like IV placement and physical examination), routine abdominal and chest ultrasound in trauma patients may improve prehospital triage of trauma patients at no extra cost. This is important, since transportation to an inappropriate, lower-level trauma center has several drawbacks. In case of under-triage, transportation of unrecognized severely injured patients to a lower-level trauma center can potentially result in unnecessary delays of treatment and thus worse outcome. Over-triage will lead to crowding in the higher-level trauma center and unnecessary high costs. Indeed, in the aforementioned recent review by van der Weide, et al including nine studies on prehospital ultrasound in trauma patients, POCUS was found to impact prehospital polytrauma management in 9.0% to 48.9% of patients; in 4.0% to 22.0% of patients, this change in management included a change in patient disposition.^3^

Point-of-care ultrasound in trauma patients seems to be reliable. Press, et al found a high specificity for the necessity of chest tube drainage for pneumothorax (99.8%).^11^ In addition, a retrospective study by another HEMS crew in the Netherlands found prehospital abdominal ultrasound to lead to a change in management in 13% of trauma patients, with a 97% specificity, 31% sensitivity, and 82% overall accuracy for hemoperitoneum.^6^ In addition, other studies showed a high level of agreement between prehospital POCUS and in-hospital ultrasound assessment and reported a change in patient management in up to 20% of prehospital trauma patients due to POCUS.^12,13^

As shown in the current study, prehospital POCUS by Dutch HEMS physicians seems to be especially beneficial for improved field triage in trauma patients and ultrasound-guided resuscitation in out-of-hospital cardiac arrest. Unfortunately, there are currently no ultrasound courses that cover both of these subjects. It is therefore suggested to develop a prehospital POCUS course specifically tailored to HEMS physicians. The curriculum of this course should aim on obtaining the necessary skills to diagnose pneumothorax, hemothorax, free abdominal fluid, and pericardial effusion/tamponade. In addition, this course should focus on the assessment of cardiac preload by determination of atrial or caval filling and assessment of ventricular function and size. As the required skills are transferable in a relatively limited amount of time, a combination of self-learning, a hands-on course, and supervised examinations may effectively improve skills.^1^ As many Western European countries now have similar prehospital systems, such a course could be highly instrumental not only for Dutch HEMS physicians, but for prehospital physicians from other countries as well.

## Limitations

This study has some limitations. While this study shows that POCUS often leads to a change in management, it is unclear if POCUS affects patient outcomes. In addition, the difference in background, training, and the frequency of using ultrasound diagnostics in practice on a daily basis is very different among the included HEMS physicians; this may have influenced the results of this study. Thirdly, in this study, the criteria for use of POCUS by the HEMS physicians were not specified, which may have led to a selection bias and could also have influenced the results of the study. Lastly, this study was conducted by a limited selection of physicians using different ultrasound equipment and in a unique prehospital emergency care system, implying that extrapolating these results to other prehospital EMS systems should be done with caution.

## Conclusion

During the study period, POCUS examination was used in 34.5% of all prehospital HEMS patients and had a therapeutic consequence in 40.7% of the prehospital patients that underwent POCUS. In trauma patients, POCUS seems to be most effective for patient triage and evaluation of treatment effectiveness. Moreover, POCUS can be of significant value in patients undergoing CPR. A tailored HEMS POCUS training curriculum should include ultrasound techniques for trauma and cardiac arrest.
